# Mechanical Properties and Frictional Wear Characteristic of Pure Titanium Treated by Atmospheric Oxidation

**DOI:** 10.3390/ma14123196

**Published:** 2021-06-10

**Authors:** Tong Chen, Shinji Koyama, Shinichi Nishida, Lihua Yu

**Affiliations:** 1 Graduate School of Science and Technology, Gunma University, Gunma 371-8510, Japan; koyama@gunma-u.ac.jp (S.K.); snishida@gunma-u.ac.jp (S.N.); 2 School of Material Science and Engineering, Jiangsu University of Science and Technology, Zhenjiang 212003, China; lhyu6@just.edu.cn

**Keywords:** oxidation, titanium, structure, bonding strength, tribological property

## Abstract

Pure titanium was treated by atmospheric oxidation, and the effect of the treatment temperature on its performance was studied. X-ray diffraction, scanning electron microscopy, wear testing, and scratch testing were used to evaluate the performance of the treated specimens. In order to evaluate the difficulty of compound formation during the different processing temperatures, Gibbs free energy was calculated. The experimental results show that the surface hardness of the sample can be improved at a certain oxidation treatment temperature. When the processing temperature is 850 °C, the surface hardness reaches the maximum value. The results of the scratch testing show that the hardened layer produced at this processing temperature has excellent peeling resistance. In addition, the wear depth and wear width are also at their minimum values at this processing temperature. Since the specimen treated at a processing temperature of 850 °C provides sufficiently high surface hardness and wear resistance in this research report, it is considered to be the optimal condition during practical application.

## 1. Introduction

Industrial pure titanium refers to dense metal titanium containing a small amount of oxygen, nitrogen, carbon, iron, and other impurities. In addition, pure titanium is an important corrosion-resistant structural material. This is because it has good corrosion resistance and excellent mechanical properties, which have led to its wide use. It is widely used in chemical equipment, power generation devices, seawater desalination devices, and ship parts. It is among the rapidly developed titanium materials. At present, titanium alloys are being developed with the aim of high performance and low cost. A notable way to achieve low cost is to conduct continuous casting and rolling of titanium, and rolling in the atmosphere involves high-temperature oxidation [1,2,3,4].

The method of forming a continuous compound layer on the metal surface is usually used to reform the wear resistance of mechanical parts. The oxidation method is widely used because of its moderate thickness, high bonding strength with the substrate, and simple operation process. Many researchers have conducted preliminary studies on the oxidation of pure titanium [5,6,7].

In these surface modification methods, heating by a furnace is the simplest and most common way to complete oxidation treatment, which can be widely used in industry. Armand et al. [8] reported that using surface treatment to treat pure titanium can appropriately improve its high-temperature oxidation resistance. Aniołek et al. [9] found that the improvement to the poor tribological properties of pure titanium after oxidation treatment is closely related to the oxide layer on the surface. Maytorena-Sánchez et al. [10] studied oxidation time and temperature separately. The results show that the effect of treatment temperature on the oxide layer is higher than that of treatment time. As is well known, titanium is active and easily reacts with oxygen. On this basis, research on the oxidation of pure titanium is extremely important. There are many researchers who have previously researched the products of pure titanium, but there are few reports on the combination of oxidation structure, mechanical properties, and tribological properties. In addition, previous researchers did not select a wide processing temperature range to systematically study the changes in the properties of pure titanium after oxidation. This article selects a wide processing temperature range to conduct a comprehensive study on the structure, mechanical properties, and tribological properties of pure titanium after oxidation. In addition, although there have been studies on the surface modification of pure titanium by oxidation, there are few studies on the oxide layer and the bonding force with the metal substrate. As is well known, titanium and titanium alloys are widely used in human bones, porcelain teeth, etc., but they often cause aging due to the low bonding force between the oxide film and metal substrate; therefore, it is particularly important to study the bonding force between these two components.

This experiment studies the oxidation behavior of industrial pure titanium at high temperatures (650–900 °C); analyzes its Gibbs free energy; and observes the oxide layer morphology, phase composition, mechanical properties, and tribological properties. The aim of this study is to synthesize an oxide layer on pure grade-2 Ti by oxidation in the atmosphere. In this research report, the method of changing the heat treatment temperature is used to study the effect of oxidation temperature on the structure and comprehensive mechanical properties of the oxide layer. In order to understand the products, we calculate the Gibbs free energy of the compound that may be generated according to the data of the NIST-JANAF thermochemistry tables. The compound formed by the reaction forms a protective layer on the surface of pure titanium to prevent serious wear loss during use. In addition, this research report also studies the effect of the processing temperature on the mechanical properties of the specimen (including the morphology of scratched grooves).

## 2. Experiment

The specimens used in this research report comprised a 50 mm × 20 mm × 5 mm plate cut from the as-received plate using fine cut machining (HS-45A2, HEIWA TECHNICA, Kanagawa, Japan). The surface of pure titanium to be treated was finished by grinding with emery paper (grade 4000). The pure titanium was heated by the air furnace to oxidation. Oxidation was carried out from 650 °C, 750 °C, 800 °C, and 850 °C to 900 °C for 2 h in the atmosphere. An X-ray diffractometer (XRD, 2200VF, Rigaku, Gunma, Japan) was used to determine the compounds formed on the surface after oxidation. The CuKα radiation working parameters of the X-ray diffractometer are a voltage of 32 kV and an anode current of 20 mA. Microstructural and morphological characteristics of oxide layers were examined using a scanning electron microscope (SEM, S-3000N HITACHI, Tokyo, Japan). An energy dispersive analyzer (EDX, SEDX-500, SHIMADZU, Tokyo, Japan) and an electron probe X-ray diffraction analyzer (EPMA, EPMA1610, SHIMADZU, Tokyo, Japan) were used to measure a cross-section of the specimen point distribution element. Surface hardness measurements were carried out on the oxidation layers of the specimens using a Vickers hardness tester (HMV-1, SHIMADZU, Kyoto, Japan) with applied loads at 0.98 N to accurately obtain the hardness of the hardened layer. Surface hardness testing was performed 5 times. The test standard of Vickers hardness testing is ISO 6507-1. In addition, the adhesion between the substrate and the diffusion layer was determined by a scratch tester (REVETEST, Anton Paar, Graz, Austria) with a Rockwell indenter tip. The normal load applied in the scratch tests gradually increased from 0 to 150 N, and the scratch speed was 10 mm/min. The scratch length was 10 mm. After scratch testing, the surface morphology of the scratch was observed by SEM. The test standard of scratch testing is ASTM C1624, ISO 20502. The tribological properties of treated specimens were elevated by ball-on-disc dry sliding testing, which was performed at room temperature. The tribometer (FPR-2000, Rhesca, Tokyo, Japan) was used with a zirconium dioxide (ZrO_2_) ball. The radius of the counter-face of the grinding ball is 2380 μm. In addition, the sliding linear speed of wear testing is 200 mm/s. The applied force (loading force during wear testing) and the test time of wear testing are 4.9 N and 3600 s, respectively. Based on this, the corresponding sliding distance is 720 m. The test standard of wear testing is ASTM G99-05. The scratch testing and wear testing were performed 3 times.

## 3. Results and Discussions

### 3.1. Structure Analysis

**Figure 1:**
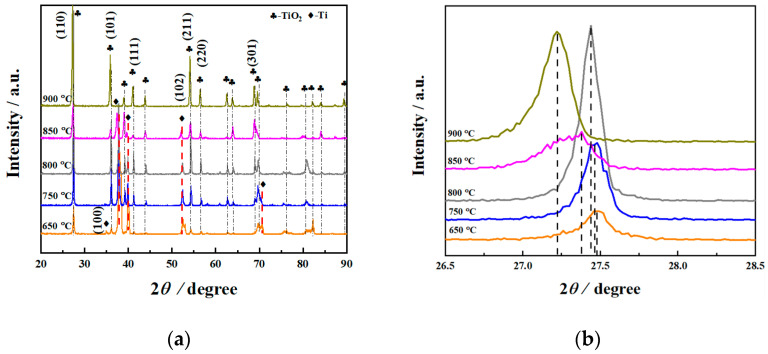
X-ray diffraction patterns (**a**) and an amplification view of the (110) peaks (**b**) of the surface of specimens with different processing temperatures.

X-ray diffraction was performed on the treated specimens at different processing temperatures, and the specific pattern is shown in Figure 1. From the XRD results (Figure 1b), it can be seen that as the processing temperature increased, the intensity of the oxide layer on the surface of the specimen increased first and then decreased. Moreover, the intensity of the diffraction peak of the matrix phase gradually decreased. In addition, the diffraction peak intensity of TiO_2_ in the (110) plane basically gradually increased with the increasing processing temperature, indicating that the growth direction is [110]. As shown in Figure 1a, it was revealed that TiO_2_ was formed on the surface. Additionally, the diffraction peak of Ti was detected at 650 °C, indicating that the oxide layer was relatively thin at this processing temperature, and the influence of the matrix peak was present. It was inferred that oxygen diffused at high temperatures and became more obvious with different processing temperatures. As is well known, under the same test conditions, the X-ray inspection depth is constant. As the thickness of the oxide layer increased, the detection of the Ti matrix peak became more difficult, resulting in a decrease in the intensity of the Ti matrix peak. It can be seen from Figure 1a that when the processing temperature was 900 °C, the Ti matrix peak was almost invisible, which may be due to the thick oxide layer at this processing temperature. The results are shown later in the present research report.

**Figure 2:**
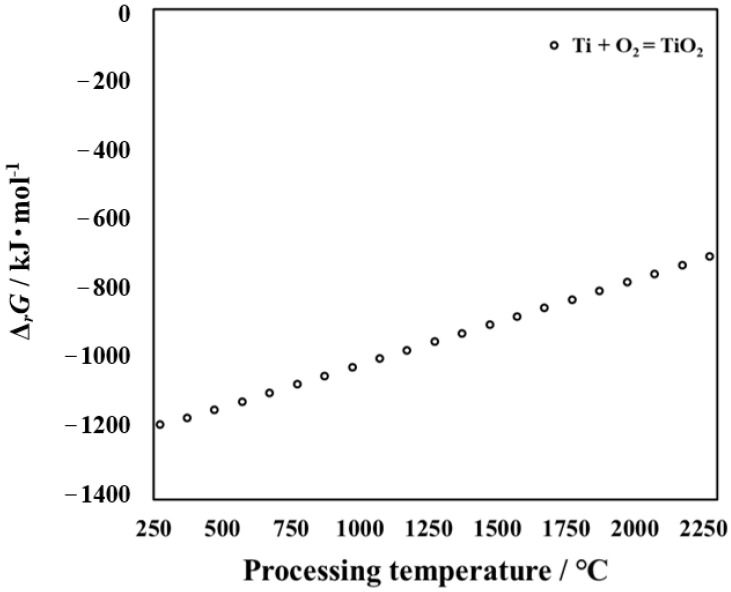
Relationship between processing temperature and Gibbs free energy Δ*rG* of reactions (1).

This research report uses NIST-JANAF thermochemical numerical data to calculate the Gibbs free energy (Δ*rG*) for oxidation reactions in the temperature range of 250 to 2250 °C [11]. The results are shown in Figure 2. The possible oxidation reaction is as follows:

Ti + O_2_ = TiO_2_ (1)

The influence of processing temperature on the Δ*rG* of reactions (1) is shown in Figure 2. When the value of Δ*rG* is less than zero, the reaction can occur. Moreover, the smaller the value of Δ*rG*, the more easily the reaction proceeds [12]. From Figure 2, it can be seen that O and Ti can react to form oxide. As shown in Figure 2, the higher the temperature, the higher the Gibbs free energy of TiO_2_ required, and the more difficult it is to generate. However, it is well known that the higher the temperature, the more active the random diffusion motion of atoms in metallic material. As a result, as the processing temperature increases, more oxygen atoms diffuse into the interior. In order to further analyze the product, its thermodynamic parameters need to be calculated. Thermodynamic calculations were carried out for the untreated titanium that may be generated during the processing in this research. Oxidation is a process in which oxygen atoms diffuse from the outside to the inside and react with titanium. With an increase in the processing time, the content of oxygen atoms on the surface and inside of the specimen is different. Conclusions can also be made from cross-sectional observation. At this time, Ti + O_2_ = TiO_2_ reaction occurs, and TiO_2_ is formed on the outermost surface. The results suggest that a large amount of TiO_2_ can be detected by XRD at high processing temperatures. The results of Gibbs free energy are consistent with those of the XRD results.

**Figure 3:**
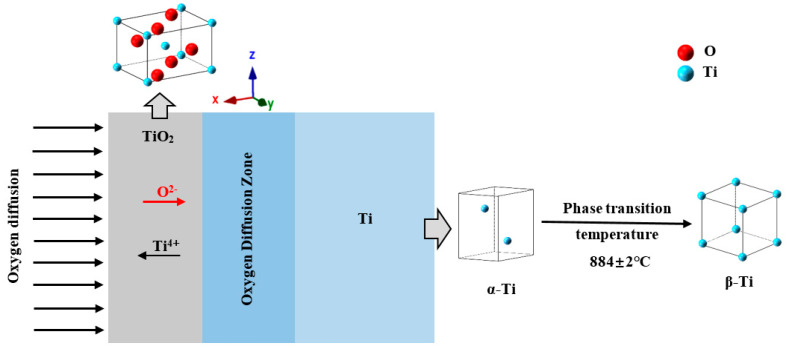
Schematic of growth of the hardened layer (TiO_2_) and the unit cells corresponding to O_2_, α-Ti, and β-Ti, respectively.

Figure 3 shows a schematic diagram of the oxide layer structure on the surface of a pure titanium sample. The unit cells in the figure are all made with the ionic radius of the substance. The red balls represent oxygen atoms, and the light blue balls represent titanium atoms. In addition, O has an ionic radius of 140 × 10^−12^ m, and Ti has an ionic radius of 68 × 10^−12^ m. It can be seen that the size of oxygen is larger than that of titanium in the unit cell of TiO_2_. During the oxidation process, oxygen atoms and titanium atoms form a rutile TiO_2_ oxide layer on the outermost layer of the sample. The space group structure is P42/mmm at room temperature. The crystallographic constants are a = 0.465 nm, b = 0.465 nm, and c = 0.297 nm [13,14]. With the extension of the oxidation time, oxygen ions diffuse through the TiO_2_ layer into the specimen, forming an oxygen diffusion zone between the Ti matrix and the TiO_2_ oxide layer [15,16]. Under high temperature conditions, the pure Ti matrix undergoes a configuration transformation from α-Ti to β-Ti, and 884 ± 2 °C is the phase transition temperature of the two structures. The schematic diagram of the lattice transformation of the transformation process is also presented in Figure 3. The space group structure of α-Ti is P63/mm, and the crystallographic constants are a = 0.293 nm, b = 0.293 nm, and c = 0.466 nm [17]. The space group of β-Ti after high-temperature phase transition is Im3-m, and the crystallographic constants are a = 0.282 nm, b = 0.282 nm, and c = 0.282 nm [18]. A uniform and continuous TiO_2_ oxide layer is formed on the surface of the pure Ti specimen, which can significantly reform the surface hardness and the wear resistance of the pure Ti specimen.

**Figure 4:**
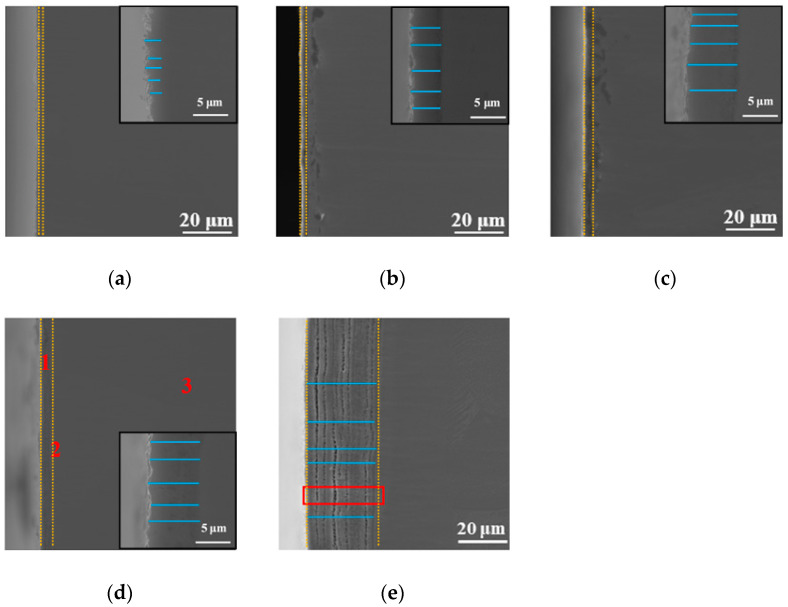
SEM micrographs of the cross-section with different processing temperatures: (**a**) 650 °C, (**b**) 750 °C, (**c**) 800 °C, (**d**) 850 °C, and (**e**) 900 °C.

**Figure 5:**
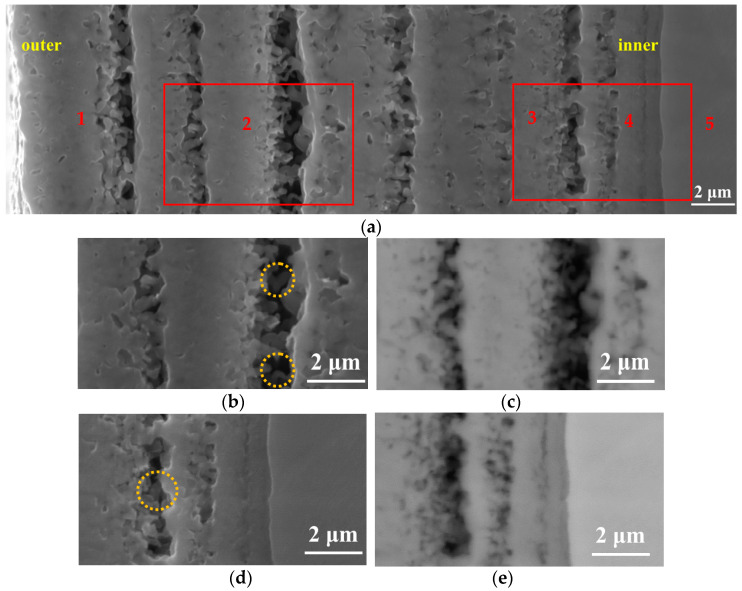
Cross-sectional observation at different locations at 900 °C. (**a**,**b**,**d**) are the morphologies observed in the SE image, and (**c**,**e**) are the morphologies observed in the BSE image.

The SEM cross-sectional morphology observation method was used to study the effect of the processing temperature on the cross-sectional morphology of the specimen, and the results are shown in Figure 4. In addition, the results of point elemental distribution (the positions marked 1, 2, and 3 in Figure 4d) are also shown in Table 1. As is well known, EDX can only be qualitatively analyzed; it cannot be quantitatively analyzed, but it can be roughly seen from Table 1 that the oxygen content gradually decreased from the outermost layer to the Ti substrate. As shown in Figure 4, the thickness and morphology of the oxidation layer varied with different processing temperatures, as observed in the vicinity of the surface. Comparing with XRD results, in each processing temperature, there was an oxide peak that differed from the Ti substrate peak. In addition, it can be seen from Figure 4 that the thickness of the oxidation layer on the surface increased as the processing temperature increased (the interval of the yellow dashed line indicates the thickness of the oxide layer at each processing temperature). Aniołek et al. [10] discussed cyclic oxidation of titanium grade 2. It was found that the processing formed an oxygen layer on the surface. In addition, it was found that the oxide layer was composed of TiO_2_. It has a different appearance from that of Ti substrate under SEM observation. This is consistent with the phenomenon observed in this study. The thickness of the cross-section oxide film in Figure 4a–e was measured by multiple lines (the blue lines are the measurement lines), and the results were 2.18 μm, 4.21 μm, 5.41 μm, 7.22 μm, and 28.18 μm, respectively. These results suggest that the thickness of this diffusion-layered structure tended to increase with increasing processing temperature. This shows that at each processing temperature, the oxide formed on the surface of the specimen. Notably, when the processing temperature increased to 900 °C, the thickness of the diffusion layer reached the maximum value. However, at this processing temperature, delamination could be clearly observed, indicating that the oxide layer was less dense. This can lead to obtaining poor mechanical properties at this processing temperature, and a specific explanation is provided later in the present research. In order to further discuss the causes of this phenomenon, we performed magnification (red rectangle position) on Figure 4e, and the results are shown later in the present research report (see Figure 5).

Point elemental distribution analysis was carried out on the specimen with the processing temperature of 850 °C, as displayed in Figure 4d. As shown in Table 1, the oxygen content from the outermost layer to the Ti substrate gradually decreased. It can be seen that oxygen gradually diffused into the interior at this processing temperature. Oxides were formed on the surface of the specimens with different processing temperatures.

The SEM micrograph of the cross-section with a processing temperature of 900 °C is shown in Figure 5. It can be seen from Figure 5 that the oxide layer formed at this processing temperature was not dense, and obvious delamination occurred. Furthermore, there was a clear gap between layers. Guleryuz et al. [19] researched the oxidation behavior of Ti-6Al-4V alloy. The results show that when the processing temperature is higher than 800 °C, a relatively thick and fragile oxide layer can be formed. The oxide layer presents a multilayer porous structure, in which linear oxidation kinetics dominate. In addition, it can also be observed that due to the oxygen affinity of titanium, it will react with oxygen in the air to form a layer of TiO_2_ on the surface. This layer can protect the substrate in various corrosive environments and prevent further oxidation and corrosion of the substrate. However, at high temperatures, the TiO_2_ layer loses its protective properties and dissolves oxygen into the titanium bulk metal. It is known from the literature that the solubility of oxygen in α-Ti is about 30 at%, and it changes little with the increase in temperature, while the solubility of β-Ti increases with the increase in temperature, reaching a maximum solubility of about 8 at% at 1700 °C [20]. The solubility of oxygen in β-Ti is very small. When TiO_2_ content reaches saturation, the growth of TiO_2_ stops, thereby forming gaps between the oxide layers.

Moreover, it can be seen from the figure that TiO_2_ grows in a columnar shape (the yellow circle position in Figure 5b,d). This is because rutile is a long columnar crystal grain, and the length of the crystal grain extends in the c direction. Oxygen atoms are most densely packed as hexagons. Titanium atoms are located in octahedral voids with a coordination number of six, and oxygen atoms are located at the center of a plane triangle with titanium atoms as the apex angle, and the coordination number is three. In the [001] direction, each octahedron has two edges shared with two adjacent octahedrons above and below, thereby forming a relatively stable octahedral chain extending along the c-axis direction, and the octahedrons sharing the corners between the chains connect the phase [21].

In order to further research the oxide layer, the cross-section of the specimen was photographed with the SEM, and we performed magnification (the red rectangle positions in the left and right represent Figure 5b,d) on Figure 5a, the results of which are shown in Figure 5b–e. Since this article only carried out oxidation treatment, in order to better distinguish the distribution of oxygen in the oxide layer, the photographs in the partial secondary electron (SE) and back scattered electron (BSE) states were collected in Figure 5, which shows the results. The difference is that Figure 5b,d were taken under SE while Figure 5c,e were taken under BSE. During the BSE observation, the darker the color appears, the more oxygen content present in this condition. In this research, it can be observed that the contrast in the image is consistent, indicating that the light element (oxygen) content in each position is roughly the same. The point elemental distribution (the positions marked 1, 2, 3, 4, and 5 in Figure 5a) result is also shown in Table 2. From Table 2, it can be seen that the oxygen content gradually decreased from the outermost layer to the Ti matrix. The oxygen content of the outer layer remained approximately the same.

### 3.2. Mechanical Properties

**Figure 6:**
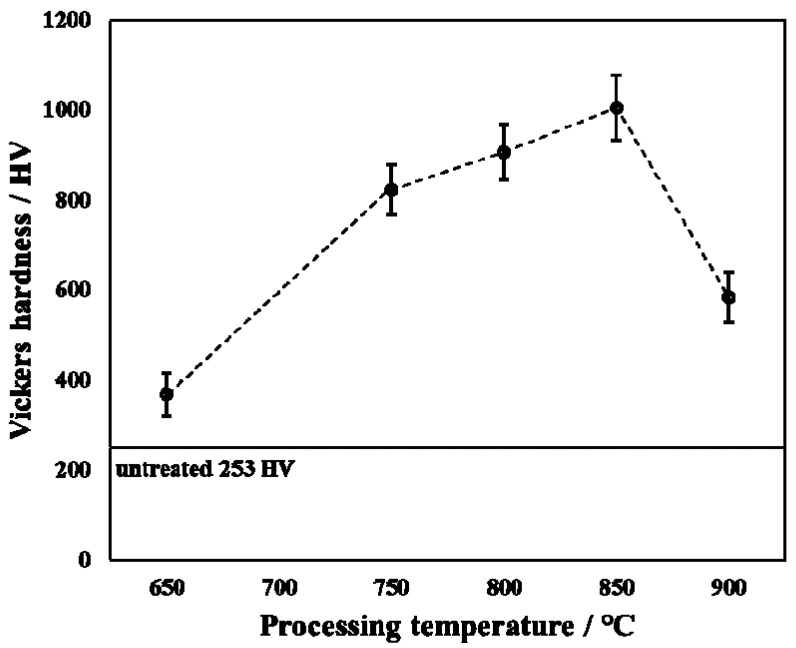
Surface hardness of untreated and treated specimens with varied processing temperatures.

Figure 6 shows the surface hardness of untreated and treated specimens with applied load at 0.98 N. From Figure 6, the surface hardness first increased and then decreased as the processing temperature increased. The surface hardness of the untreated specimen was about 253 HV, whereas the surface hardness reached a maximum value of 1005 HV at 850 °C. Sivakumar et al. [22] revealed that the hardness of TiO_2_ is about 800 HV. This may be due to the presence of TiO_2_ causing the surface hardness to increase in the present research. However, a few of the surface hardness values measured (such as 650 °C) in this present research are less than those in the Ref. [22]. When the processing temperature was at 650 °C, the thickness of the oxide layer was small, which in turn made the Ti substrate affect the result of the surface hardness measurement. However, when the processing temperature increased to 900 °C, although the thickness of the hardened layer was relatively thick, its density was poor. In addition, although the content of TiO_2_ in the product was relatively high (in XRD results, TiO_2_ diffraction peak intensity is higher), which should have increased the surface hardness of the treated specimen, the delamination of the surface oxide layer resulted in no significant surface hardness increase. 

According to the measured surface hardness, the specific indentation depth is as shown in Table 3. From Table 3, it can be observed that at the processing temperature of 850 °C, the indentation depth reached its minimum value. Additionally, the indentation depth (2.74 μm) at this processing temperature was smaller than the thickness of the hardened layer (7.22 μm). From Table 3, it can be seen that when the processing temperature was increased to 650 °C, the indentation depth was at its largest, and this indentation depth (4.54 μm) was greater than the thickness of the hardened layer (2.18 μm), so the measured surface hardness was greatly affected by the Ti substrate, resulting in the surface at this processing temperature to have low hardness. When the processing temperature was increased to 900 °C, although the thickness of the hardened layer (28.18 μm) was very large, the delamination of the hardened layer could be clearly observed, as shown in Figure 5. This is the reason for the low surface hardness at this processing temperature.

**Figure 7:**
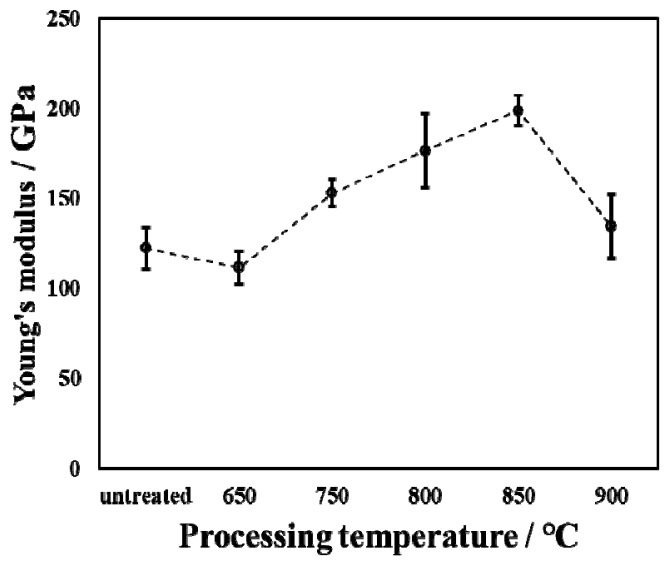
Young’s modulus of untreated and treated specimens by oxidation with varied processing temperatures.

Figure 7 shows Young’s modulus and force–displacement curves of the untreated and treated specimens during the oxidation process with varied temperatures. The loading time, holding time, and unloading time of the indentation testing are all 5 s. As is well known, the ability of materials to resist elastic deformation can be characterized by measuring the value of Young’s modulus. The Young’s modulus of the untreated specimen is approximately 122 GPa. The Young’s modulus of the treated specimen is higher than that of the untreated specimen. However, when the processing temperature was 650 °C, Young’s modulus value was lower than that of the untreated specimen. This is because at this processing temperature, a small amount of oxide was generated on the surface, and the oxide layer was very thin, which was greatly affected by the Ti substrate during the measurement, thereby reducing Young’s modulus. Anderson et al. [12] showed that the Young’s modulus of TiO_2_ is about 250 GPa. In this research report, the increase in Young’s modulus is due to the presence of titanium dioxide. However, the Young’s modulus measured in this experimental report is less than that in Ref. [12]. This is due to the fact that the thickness of the oxide layer is relatively thin, and the Ti matrix has a greater influence when measuring Young’s modulus. As the processing temperature increases from 650 °C to 900 °C, Young’s modulus first increases and then decreases. When the processing temperature was increased to 850 °C, the Young’s modulus reached its maximum value. This represents the fact that the ability to resist elastic deformation is the greatest at this processing temperature.

**Figure 8:**
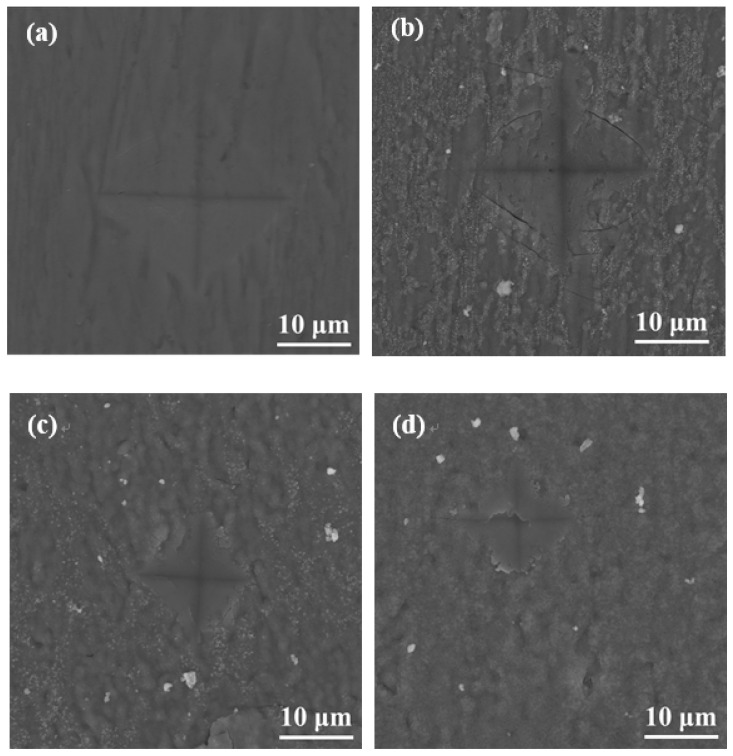

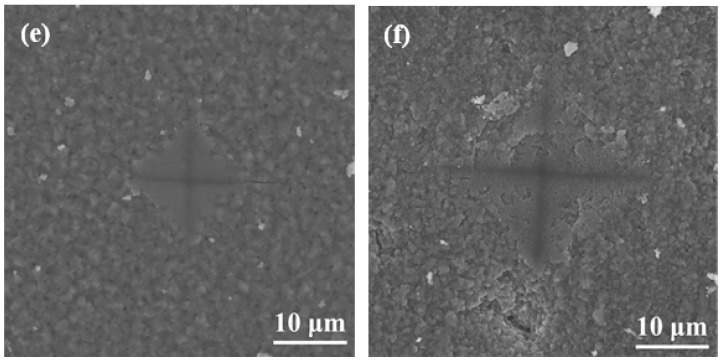
SEM micrographs of the indentation with varied processing temperatures of (**a**) untreated specimens and those treated at (**b**) 650 °C, (**c**) 750 °C, (**d**) 800 °C, (**e**), 850 °C and (**f**) 900 °C.

**Figure 9:**
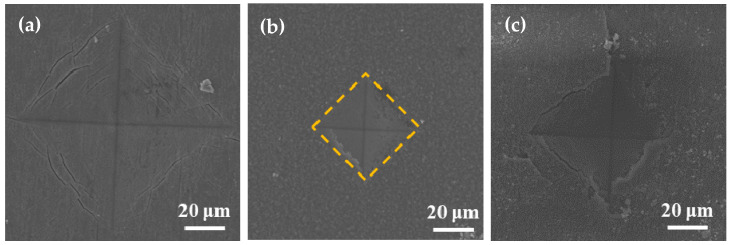
SEM micrographs of the indentation with varied processing temperatures of (**a**) 650 °C, (**b**) 850 °C, and (**c**) 900 °C.

During surface hardness testing, the residual stress on the specimen surface interacts with the force applied by the indenter, which affects the actual indentation morphology. Based on the surface hardness indentation morphology, the properties of the residual compressive or tensile stress on the surface of the specimen can be qualitatively determined. The results of this test provide assistance in explaining the wear resistance of the surface hardened layer. Notably, it contributes to explaining the fatigue resistance of the surface hardened layer. Appropriate residual compressive stress on the surface is beneficial in improving wear resistance. For the same position on the specimen, the multiple reciprocating motions of wear testing are equivalent to those of fatigue testing. According to Ref. [23], when measuring surface hardness, the residual stress affects the shape and size of the specimen indentation. When there is tensile stress on the specimen surface, dents appear around the dents, and when there is compressive stress, protrusions appear around the dents. Figure 8 shows the SEM micrographs of the indentation morphology of the experiment at various processing temperatures. As shown in Figure 8, it is assumed that the residual stress of the untreated specimen is approximately zero. According to the shape of the indentation, the type of stress in Figure 8 can be determined. The internal stresses in Figure 8b are approximately equal to 0, and those in Figure 8c,d are compressive stresses. It can be seen from Figure 8c,d that as the processing temperature increased, the compressive stress was also different. It can be seen from Figure 8 that under the action of compressive stress, cracks (the yellow circle position) of varying degrees appeared. In addition, when the processing temperature was increased to 900 °C, it can be seen from Figure 8 that under the same loading force, the size of the indentation was obviously greater than 850 °C, which also indicates that the surface hardness of the specimen was lower. In addition, no cracks were found at this processing temperature. This is due to the porous layered structure at this processing temperature, which alleviates the effect of stress. The indentation morphology of the treated specimens at 650 °C, 850 °C, and 900 °C was observed under a high loading force (9.8 N) in order to further analyze the morphology of the indentation. The results are shown in Figure 9. When the processing temperature was increased to 650 °C, combined with the results of Young’s modulus, it can be seen that the resistance to deformation at this processing temperature was weak (see Figure 7). It can be seen from Figure 9a that in this condition, the shape of the indentation was almost a square, indicating that the stress was very small in this condition, almost close to zero. When the processing temperature was increased to 850 °C, in order to facilitate the analysis of the indentation morphology, the four corners of the indentation were connected with a yellow dashed line. The result is shown in Figure 9b, where the indentation is curved toward the center of the yellow dashed line at this processing temperature, indicating that there was compressive stress in this condition. According to the results in Figure 7, it can be seen that Young’s modulus at this processing temperature reached its maximum value, indicating that the resistance to elastic deformation was strongest in this condition. However, no cracks can be observed in Figure 9b, indicating that the compressive stress in this condition is relatively large. Moreover, its ability to resist deformation is strong, which reduces the influence of external forces on the specimen. When the processing temperature was increased to 900 °C, it can be seen from Figure 9c that more serious cracks occurred. Because it was under the same loading force, the internal stress of the specimen was relatively large, and the specimen under this condition did not have the ability to resist elastic deformation (Young’s modulus was low), causing cracks to occur. In addition, it can be seen from Figure 4 that delamination of the hardened layer at this processing temperature occurred. Under the same loading force, compared with other conditions, it was more easily damaged, which led to the peeling of the hardened layer.

**Figure 10:**
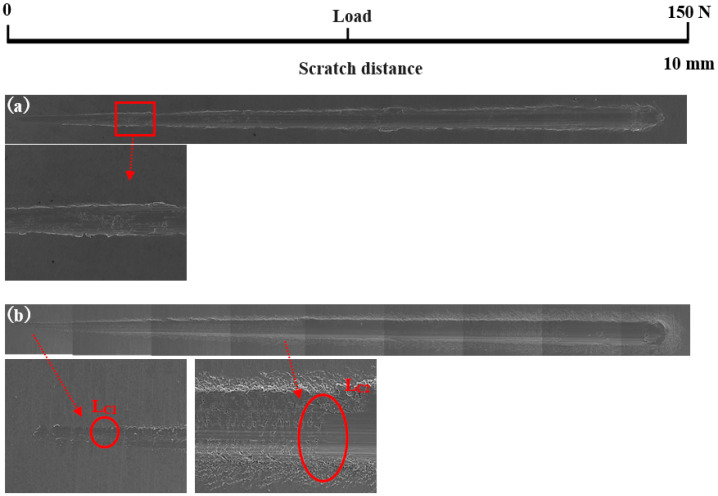

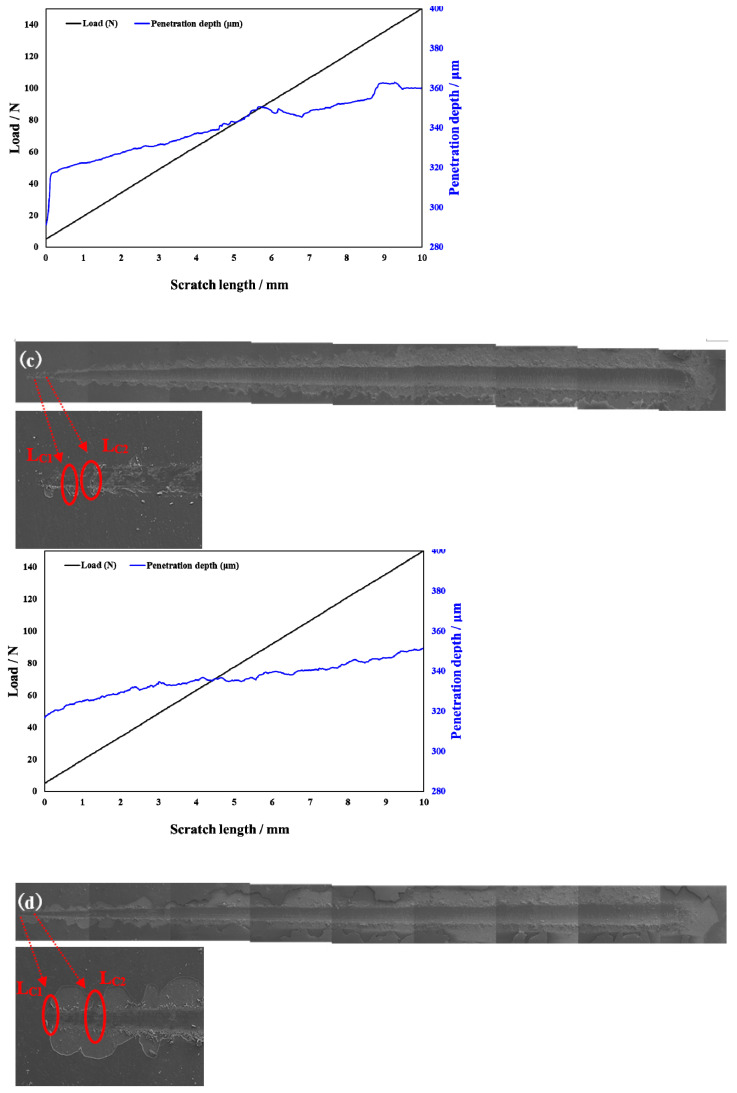

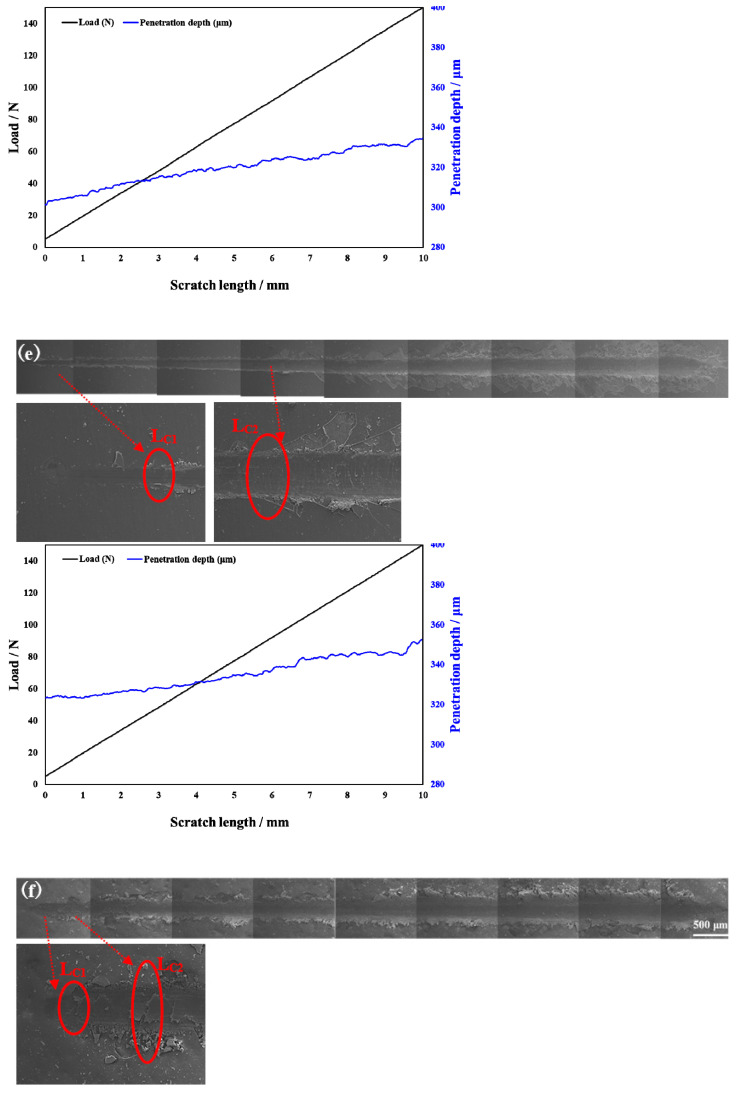

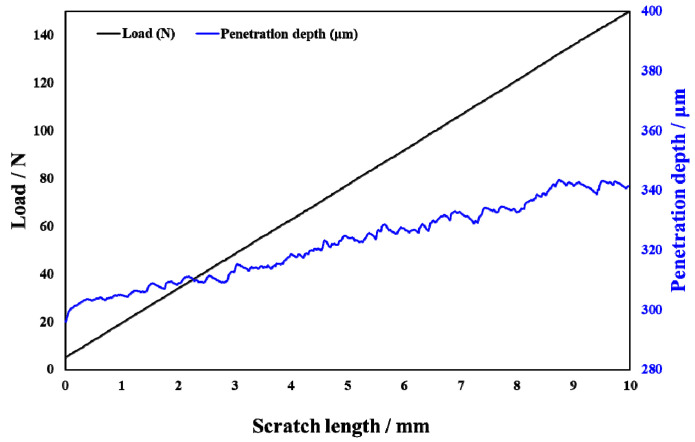
Scratch groove morphology and curve of force and indentation depth with scratch length of the specimens treated at various processing temperatures: (**a**) untreated and treated at (**b**) 650 °C, (**c**) 750 °C, (**d**) 800 °C, (**e**) 850 °C, and (**f**) 900 °C.

Scratch testing was performed on hardened layers to quantitatively determine their cohesion and adhesion strength. Figure 10 presents the SEM image of the scratch track and the corresponding scratch loads and distances. The curve of force and indentation depth with the scratch length are also shown in Figure 10. From this curve, it can be seen that the depth of the grooves increased with the increasing load from 0 to 150 N. The result of the scratch test for the untreated specimen is shown in Figure 10a. Comparing the results of the untreated specimen with those of the specimens treated at different processing temperatures, there were more cracks, chippings, and other defects around the scratched surface of the hardened layer, and there was also a local oxidation-hardened layer that could be peeled off, indicating that the bonding strength of the hardened layer/substrate substantially decreased. At 750 °C, 800 °C, and 900 °C, the peeling of oxidation-hardened layers was particularly noteworthy. This is because high-hardness, single-element materials undergo brittle fracture when the load is large (greater than the elastic limit of the material) [24]. When the processing temperature was 650 °C, due to the low surface hardness of the specimen, obvious hardened layer peeling did not easily occur under external loading, but obvious cracks appeared in the initial stage of loading. This is due to the fact that the oxidation-hardened layer was relatively soft and could not withstand a large load, causing it to lose effectiveness. Moreover, because the surface hardness of the hardened layer was relatively small, no large peeling occurred, but cracks developed outward. When the processing temperature was increased to 850 °C, the hardened layer did not peel off as the loading force reached about 60 N, and the adhesive between the hardened layer and the substrate was better at this processing temperature. With the increasing loading force, cracks that expand outward appeared in the hardened layer, and peeling gradually occurred.

In Figure 10, *L*_C1_ and *L*_C2_ represent the cohesion and bonding strength of the treated specimens, and the specific morphology is shown in the enlarged image. *L*_C1_, *L*_C2_, and *CPR_S_* values treated with different processing temperatures are shown in Table 4. From Table 3, when the processing temperature was increased to 850 °C, both the *L*_C1_ and *L*_C2_ also increased to the value of 8.5 N and 60.0 N, respectively. It can be observed that even though the initial incorporation of O into the hardened layer improved the cohesive strength, a further increase in the content of oxide decreased the cohesive strength. It can be seen from Figure 10 that with the increase in O content, the *L*_C2_ value showed a trend of increasing first and then decreasing. Usually, the critical loads *L*_C1_ and *L*_C2_ can also be used to evaluate the toughness of the hardened layer [25,25,26,27,28]. Zhang et al. [29] proposed that the scratch crack propagation resistance parameter *CPRs* = *L*_C1_(*L*_C2_ − *L*_C1_) can also be used to evaluate the toughness of the hardened layer after scratch testing. Using this formula for calculations, the *CPR* values under various conditions in this research report are shown in Table 4. When the processing temperature was increased to 850 °C, the treated specimens’ *CPR* values were higher than those of the specimens treated at other temperatures. The improvement in *CPR* value at the processing temperature of 850 °C was due to the formation of oxides with a high Young’s modulus. The hardened layer with the processing temperature at 850 °C exhibited the highest toughness after the scratch test with a value of 438.0. Although the value of *CRP_S_* at 650 °C were also high, the surface hardness at this processing temperature was low. This shows that at this processing temperature, the surface hardness was low, and the resistance of the specimen to plastic deformation was lower still. This led to the mechanical properties of the specimen being relatively poor.

### 3.3. Tribological Properties

The average wear width and wear depth of specimens treated with various processing temperatures after wear testing are shown in Table 5. The standard deviation values are also shown in Table 5. As shown in the table, the untreated specimen’s wear depth was about 58.0 μm. The wear depth of specimens after oxidation was less than that of the untreated specimen. With the increase in processing temperatures, the wear depth and the wear width decreased and then gradually increased. When the temperature was raised to 850 °C, the specimen achieved its optimum wear resistance. The wear width and wear depth reached the minimum values of 918 μm and 10.7 μm, respectively, in this condition. The reason for this result is that the surface hardness under this condition reached the maximum value. The higher surface hardness led to a smaller wear depth of the hardened layer. In addition, based on the measurement of the standard deviation values, when the processing temperature was 850 °C, the standard deviation in this condition was the smallest, indicating that the measured data in this condition are more accurate than those in other conditions. The wear depth can be calculated by Equation (2) by supposing that the grinding ball was not worn during wear testing [30].

(2) $$d=r-{\left[{\left(r\right)}^{2}-{\left(\frac{w}{2}\right)}^{2}\right]}^{1/2}$$

In Equation (2), the *r* is 2380 μm.

*d* is the calculated value of the wear depth, and *w* is the measured value of the wear width.

As shown in Table 5, the calculated value of the wear depth under each treatment condition is greater than the actual measured value. It can be seen from these results that, in addition to the abrasion of the hardened layer, abrasion of the grinding ball was also caused during wear testing. The difference between the actual value and the calculated value is the wear of the grinding ball. The reason for these differences is the change in wear mechanism—it changed from mechanical wear to abrasive wear.

**Figure 11:**
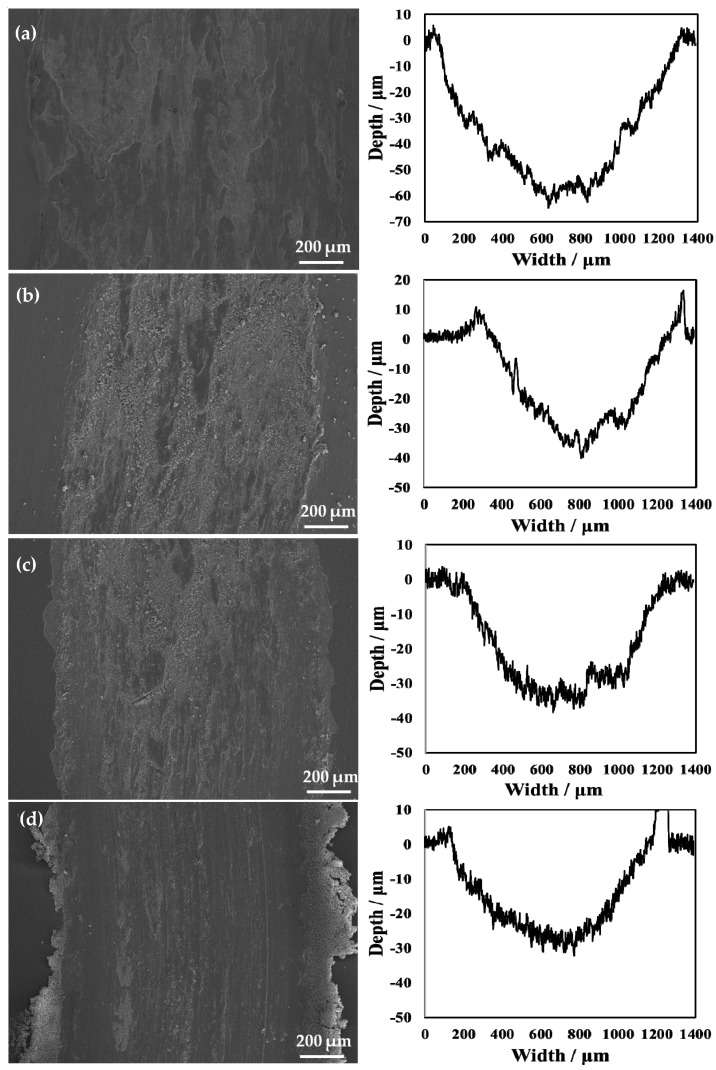

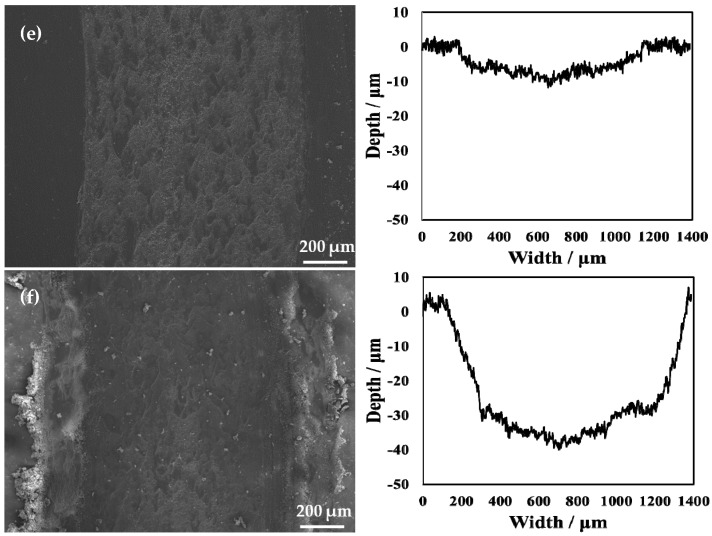
Wear scars and 2D profilometric view after wear test of the (**a**) untreated specimen and that treated at (**b**) 650 °C, (**c**) 750 °C, (**d**) 800 °C, (**e**) 850 °C, and (**f**) 900 °C.

The SEM micrographs and 2D profilometric view of both untreated and treated specimens with different processing temperatures are shown in Figure 11. From the 2D profile showing the depth of wear, it can be seen that the oxidation treatment of pure titanium reformed its wear resistance. Notably, when the processing temperature was increased to 850 °C, it can be clearly seen that the width of the wear scar became narrower. However, the untreated specimen showed a wide wear track. Moreover, the average wear depth of the wear track is 58.0 μm for the untreated specimen. The rough 2D wear track is shown in Figure 11a. The untreated specimen shows a larger and deeper wear track compared to that of the treated specimens. During the wear test, the surface of the untreated specimen was severely smeared. This indicates that it had severe plastic deformation. In addition, it was found that when the processing temperature was increased to 650 °C, the wear track was deep, but it was lower than that of the untreated specimen. This is because the generated hardened layer plays a role in protecting the matrix and reduces the wear amount in wear testing. In addition, when the processing temperature was increased to 750 °C, the hardened layer was very thin. In addition, it can be seen from the results of the scratch testing that the binding strength at this processing temperature was relatively low, and particles that increase wear were generated during wear testing. When the processing temperature was increased to 850 °C, due to the better resistance to deformation of the hardened layer, no obvious deformation and smearing effects of the worn surface were observed, as shown in Figure 11. This resulted in a significant reduction in the wear depth. This is because the hardened layer plays a role in protecting the substrate. Notably, for the wear resistance of materials, due to the presence of the hardened layer, the adhesion of the soft Ti matrix and the abrasive wear resistance increased. Maytorena-Sánchez et al. [31] also indicated that the amount of wear was lower at a processing temperature of 850 °C. However, when the processing temperature was increased to 900 °C, the wear of the hardened layer intensified. This is because there were a considerable amount of oxides on the surface at this processing temperature, which led to the decrease in toughness of the specimen. Consequently, the oxide layer was easily broken. In addition, since the hardened layer at this processing temperature had a porous structure, there were gaps between the layers, which were easily peeled off under the action of an external loading force. During wear testing, the oxide peeled off from the substrate and entered the space between the specimen and the grinding ball. The peeling oxide moved on the surface of the specimen under the drive of the grinding ball, resulting in abrasive wear. It can also be observed from Figure 11f that there were many abrasive particles on the surface. At this processing temperature, due to a thick oxide layer, the wear of the specimen was the most serious at the selected processing temperatures, and the wear amount reached its maximum value.

## 4. Conclusions

The effects of the formation of oxide layers on grade-2 pure Ti with different oxidation temperatures in the atmosphere are investigated in this present research report. This research report characterizes the structural, mechanical, and tribological properties of grade-2 pure Ti after oxidation under different conditions. The specific conclusions are as follows:

(1) Titanium oxide can be formed at all processing temperatures, where the content of the oxide depended greatly on the processing temperature.
(2) The increase in surface hardness is due to the diffusion of oxygen to Ti. When the processing temperature is 850 °C, the surface hardness reaches the maximum value.
(3) When the processing temperature is 850 °C, the adhesiveness between the hardened layer and the substrate is good.
(4) During the wear test, the untreated specimen undergoes severe plastic deformation, causing the surface to be severely smeared. The wear depth and width reached their minimum values at the processing temperature of 850 °C. Due to the high surface hardness, the oxidation process used in this research greatly improves the wear resistance of Ti.

## Figures and Tables

**Table 1:** Element content of oxygen on the surface with the processing temperature at 850 °C (analyzed by EDX).

Locations	Location 1	Location 2	Location 3
Elements (at%)	12.80	3.27	0.82

**Table 2:** Element content of oxygen and titanium on the surface with the processing temperature at 900 °C (analyzed by EPMA).

Elements (at%)	Point 1	Point 2	Point 3	Point 4	Point 5
Ti	61.46	58.48	61.20	73.88	80.43
O	38.54	41.52	38.80	26.12	19.57

**Table 3:** Indentation depth with varied processing temperatures.

Processing Temperature/°C	Untreated	650	750	800	850	900
Indentation depth/μm	5.47	4.54	3.03	2.89	2.74	3.60

**Table 4:** *L_C_*_1_, *L*_C2_, and CPR values of specimens treated with various processing temperatures.

Processing Temperature/°C	*L*_C1_/N	*L*_C2_/N	*CPR_S_ L*_C1_(*L*_C2_ − *L*_C1_)
650	10.8	67.1	608.0
750	5.7	8.8	17.7
800	5.3	6.8	7.95
850	8.5	60.0	438.0
900	7.9	10.7	22.1

**Table 5:** Average wear width and wear depth of specimens with different processing temperatures after wear testing.

Processing Temperature/°C	Wear Width/μm	Wear Depth/μm	
		Measured	Calculated
untreated	1240 ± 107.3	58.0 ± 5.9	82.2
650	1097 ± 52.9	38.6 ± 4.9	64.1
750	1137 ± 75.4	36.4 ± 8.0	68.9
800	1178 ± 93.4	29.8 ± 4.8	74.0
850	918 ± 45.6	10.7 ± 3.0	44.7
900	1164 ± 33.6	39.4 ± 3.0	72.3

## Data Availability

Data are available on request to the corresponding author.
